# Building a database for energy sufficiency policies

**DOI:** 10.12688/f1000research.108822.2

**Published:** 2022-07-04

**Authors:** Benjamin Best, Johannes Thema, Carina Zell-Ziegler, Frauke Wiese, Jonathan Barth, Stephan Breidenbach, Leonardo Nascimento, Henry Wilke

**Affiliations:** 1 Wuppertal Institut für Klima, Umwelt, Energie gGmbH, Döppersberg 19, Wuppertal, D-42103, Germany; 2 Öko-Institut e.V., Büro Berlin, Borkumstraße 2, Berlin, D-13189, Germany; 3 Technische Universität Berlin, Straße des 17. Juni 135, Berlin, D-10623, Germany; 4 Europa-Universität Flensburg, Auf dem Campus 1, Flensburg, D-24943, Germany; 5 ZOE. Institute for future-fit economies gUG, Norbertstr. 31, Cologne, D-50670, Germany; 6 GermanZero e.V., Geschäftsstelle Berlin, Franklinstraße 27, Berlin, D-10587, Germany; 7 NewClimate Institute, Waidmarkt 11a, Cologne, D-50676, Germany; 8 Wageningen University and Research, P.O. Box 9101, Wageningen, 6700 HB, The Netherlands

**Keywords:** energy demand, sufficiency policy, behavioural change, energy descent, socio-ecological transformation, policy database

## Abstract

Sufficiency measures are potentially decisive for the decarbonisation of energy systems but rarely considered in energy policy and modelling. Just as efficiency and renewable energies, the diffusion of demand-side solutions to climate change also relies on policy-making. Our extensive literature review of European and national sufficiency policies fills a gap in existing databases. We present almost 300 policy instruments clustered into relevant categories and publish them as "Energy Sufficiency Policy Database". This paper provides a description of the data clustering, the set-up of the database and an analysis of the policy instruments. A key insight is that sufficiency policy includes much more than bans of products or information tools leaving the responsibility to individuals. It is a comprehensive instrument mix of all policy types, not only enabling sufficiency action, but also reducing currently existing barriers. A policy database can serve as a good starting point for policy recommendations and modelling, further research is needed on barriers and demand-reduction potentials of sufficiency policy instruments.

## Introduction

Sufficiency is a potentially low-cost, fast and socially just mean of global greenhouse gas (GHG) mitigation. It is linked to multiple benefits for human well-being. ^1^ The understanding of sufficiency varies across disciplines and discursive spheres; in relation to energy, sufficiency is the strategy that aims at achieving absolute reductions in the use of energy-based services. ^2^ Energy sufficiency, renewable energies and energy efficiency are all methods to reduce emissions associated with energy use. Renewables substitute fossil fuels, efficiency means finding ways to reduce losses by improving the input/output relation, and sufficiency equals avoiding/shifting energy services altogether.

Just as renewable energy and efficiency technologies, energy sufficiency is enabled and promoted by policy action. It is a genuine field of policy making, a case for regulatory frameworks and infrastructures, much beyond micro-level individual behaviour changes. ^1^^,^^3^^–^^6^ We differentiate three sufficiency types to categorise policy instruments by whether they are aimed at a clear *avoid* strategy, i.e. a reduction of services; at *shift*, i.e. substituting energy intensive practices by less energy-intensive ones; or in *general* supporting reductions and substitutions. ^[^ ^1^ ^]^ It is important to note that energy use is not an end in itself, but a mean to satisfy human needs (mobility, security, connectivity), which is why the literature speaks of *energy services*.

### Information gap: a sufficiency policy database

The need for a sufficiency policy database has been formulated by various researchers and ministerial representatives, e.g. Toulouse *et al.* ^7^ and Zell-Ziegler and Förster. ^8^ Sufficiency is underrepresented in climate change mitigation policy and energy and climate scenarios so far ( *e.g.* Samadi *et al.* ^9^). A database on energy sufficiency could serve as a starting point for the collection of policy instruments, their assessment in a structured format, as precondition for further research and policy consulting and to complement the discussion on mitigation policy options.

Such database should provide information on good practices, implementation examples and insights on emission and energy reduction potentials. An openly accessible database can collect findings, ideas and examples and make them better accessible to policy makers than’hidden’ in e.g. a project report.

When consulting existing climate end energy policy databases, for example International Energy Agency’s (IEA) Policy Database ^10^ and European Environment Agency’s (EEA) Database on Greenhouse Gas Policies and Measures in Europe, ^11^ it is striking that demand-side solutions to climate change and especially sufficiency options are underrepresented. Many sufficiency-minded bottom-up initiatives are included in the database on Transformative Social Innovations ^12^ hosted by Dutch Research Institute For Transition (DRIFT), but this database does not focus on policy instruments. The NewClimate Institute’s Climate Policy Database ^13^ and the Mesures d’Utilisation Rationnelle de l’Energie (MURE) database on energy efficiency measures ^14^ include sufficiency and/or the reduction of energy service demand in their search masks. However, the scope of these databases is different to our purpose: the Database of NewClimate aims at tracking climate action worldwide and thus focuses on mitigation plans and decarbonisation roadmaps rather than on specific policy instruments. The MURE database explicitly considers sufficiency policies and indicators since spring 2021, ^15^ including detailed descriptions and quantification, but only for policies that are already implemented, which limits the openness for new proposals. Another database is the Policy Ideas Database for Sustainable Prosperity from the Institute for Future-Fit Economies (ZOE). ^16^ The ZOE-database is not a classical database, but a narrative and graphical visualisation, however, it is a valuable source for visionary cross-sectoral policies.

## Methods

This section briefly describes the structure of the database, starting with sources for entries. To improve accessibility, and to allow for filtering, clustering and potential further analyses, we propose a number of structuring categorisations including information on sectors, goals, target indicators, policy instruments and sufficiency types. Moreover, we outline our internal review and harmonisation process and the technical setup of the database.

### Data sources

Policy instruments are mainly extracted from existing literature and databases using the classifying parameters outlined below. Two key sources are first an extensive and detailed report describing policy measures for a “German Zero” emissions scenario until 2035 ^17^ and second a list of policy measures extracted from all European National Energy and Climate Plans (NECPs) published by Zell-Ziegler *et al*. ^2^ and condensed to merge instruments described by various NECPs. This is complemented by databases such as the Sustainable Prosperity Database ^16^ and by further literature ^18^^–^^30^ as well as exchange with experts (advisory board meeting of the research group EnSu in September 2020). For a full list of references, see the respective tab in the database (please see underlying data). ^31^

### Build-up of database - structuring the data

To allow for the analysis of policy instruments, we structured the database along several key categories:

**Sector** includes information on the covered sector: agri-food, buildings, industry/production, transport, LULUCF (land use, land-use change and forestry), energy and cross-sectoral (for overarching policies targeting various sectors).

**Goal/policy strategy** describes what the respective policy aims at, while

**Measure/action** explains the more concrete changes envisaged to achieve the policy goals and the mitigation target.

**Indicator** lists quantifiable units to be used to estimate the effect of the policy instrument/policy.

**Sufficiency type** categorises instruments according to the strategies avoid, shift or generally supporting.

**Policy instrument** lists the specific policy instrument from the respective source that is intended to lead to change and the description gives more details on the specifications of the instrument.

**Instrument type** categorises the policy instrument types according to UNFCCC categories, ^32^ adding one for "not specified", where actions are listed in sources but respective instruments are not specified.

**Time horizon impact/implementation** rough estimate of time until possible implementation and time between implementation and impact.

**References** are included with a link and page reference to facilitate retrieval of original sources.

The latest version of the database is available under https://energysufficiency.de/policy-database/.

As the database will be further developed, additional categories may be included such as quantification (parameter settings as an input for system modelling), impact chains (as a guidance for sector-specific modelling), extension beyond Germany/Europe, push/pull measures, ^33^ governance levels, ^34^^,^^35^ policy interactions, and whether they constitute a sufficiency promotion or reduction of barriers as well as metadata.

### Internal review

In order to increase consistency and interrater reliability of the database and to avoid duplicate entries, the data collection and cleaning process was organised with multiple internal review loops:

• Assignation of main sector responsible to each sector (out of the four first authors)
• Initial policy collection by main sector responsible
• Assignation of reviewer to each sector (out of the four first authors), review of all entries including: clarity of entries, correct coding, plausible entries (esp. goal/policy strategy, measure/action, indicator, instrument type)
• Bilateral coder meetings for clearance of divergences where necessary
• Loops of coder-meetings to sort out inconsistencies and establish a common understanding and coding procedure
• Where multiple policy instruments were listed, prioritisation on main instrument or splitting of policy in respective separate instruments (rows)
• Harmonisation of goals and indicators within and across sectors

### Build-up of database

The database needs to meet the following requirements in order to be of highest utility and used most efficiently:

• Online availability with open access
• Clear design and easy navigation through the dataset
• Possibility to filter and search for keywords
• Possibility to insert additional information on policies (description as floating text)
• Possibility to include attachments (e.g. sufficiency policy impact chains, planned to be developed)
• Possibility to download the data

Furthermore, we plan to include interfaces for feedback and crowd-sourcing of new policies.

In order to find a software solution that may deliver on these aspects we exchanged with the hosts of comparable policy databases and with persons from our networks and a person from the EnSu project’s advisory board ^[^ ^2^ ^]^.

## Results

**Figure 1.**
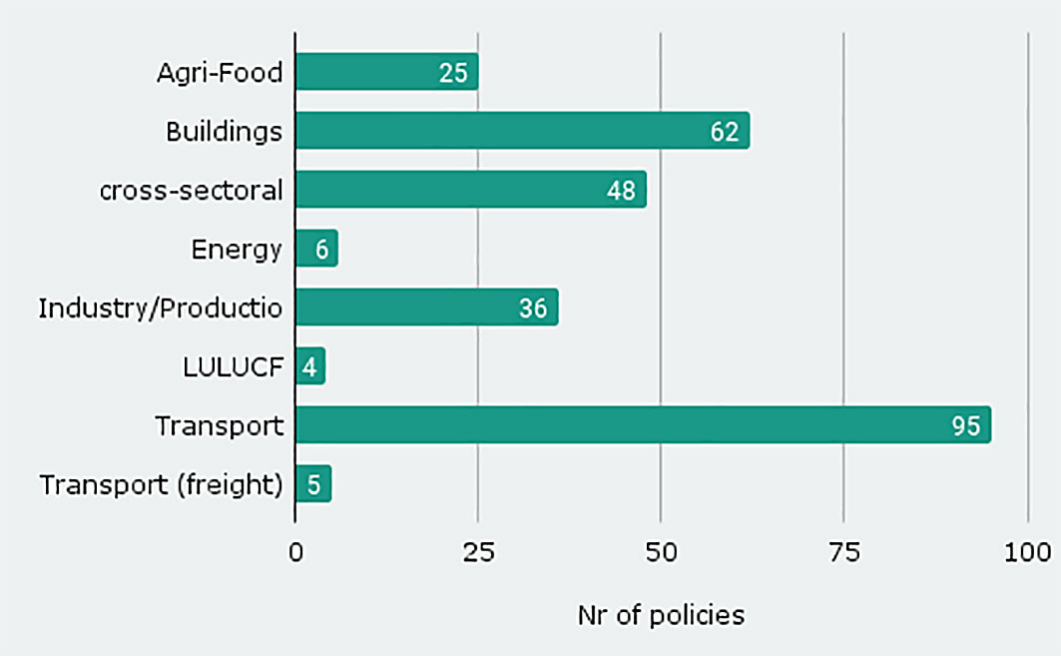
Number of policy instruments by sector.

The database version published with this article contains 281 policies from seven sectors ( Figure 1 shows freight transport separately). One third of these are from the transport sector, followed by 62 policies in the building sector and 48 cross-sectoral measures that address more than one sector. Some provide indirect connections with sufficiency measures such as reductions of subsidies for fossil fuels. The sources we analysed contained only very few sufficiency measures for the LULUCF (4) and energy (6) sector. The sufficiency measures in the energy sector concentrate on the overall policy goal ‘reduce energy consumption’ with such instruments as e.g. ‘subsidise energy savings’. ^28^

**Figure 2.**
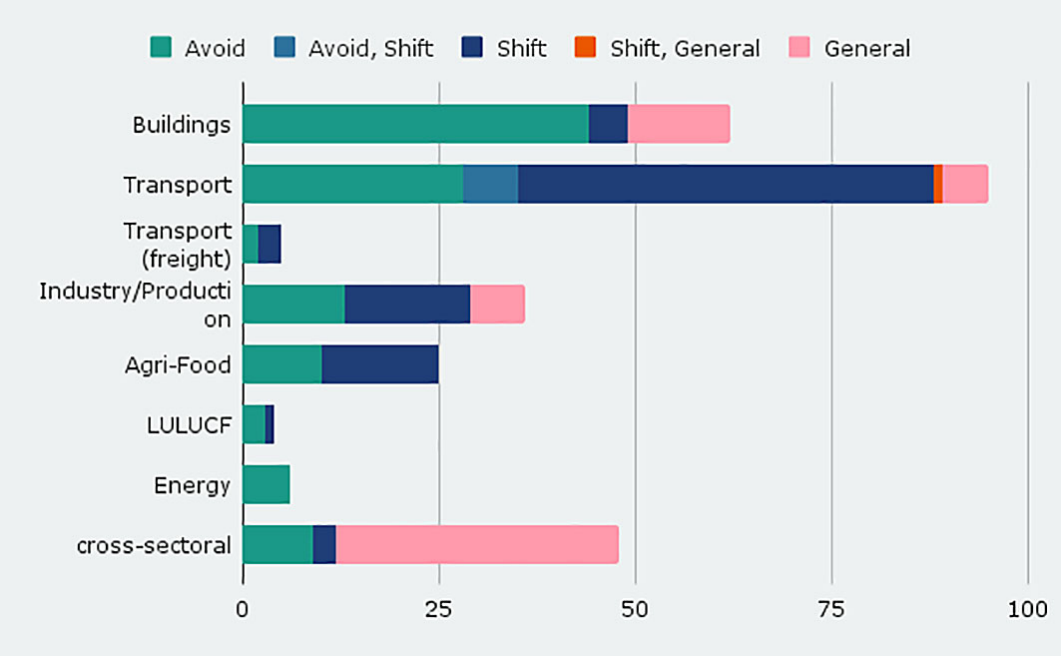
Number of policy instruments by sector and sufficiency type.

We include modal shift measures in the transport sector, labeled as sufficiency type shifting from high-energy to lower-energy modes (shift). Of the 95 transport entries, 56% aim at mode shift from cars to public transport and active modes (walking, cycling) (see Figure 2). In the transport sector, shift policies dominate, indicating that this is a key policy strategy for transport. Somewhat less common is the reduction of necessary trips and thus traffic (avoid), e. g. through support of teleworking or city planning to reduce distances between points of interest. Shift policies also dominate in the industry and agri-food domain.

In the building sector, sufficiency instruments of the ‘avoid’ type dominate in our database so far, aiming at an efficient use of living space and development of the existing building stock, focusing on quality of living and by that reduce overall living space and required land sealing. In the industry/production sector, avoid measures target at enhancing product lifetimes by increasing reparability and durability.

**Figure 3.**
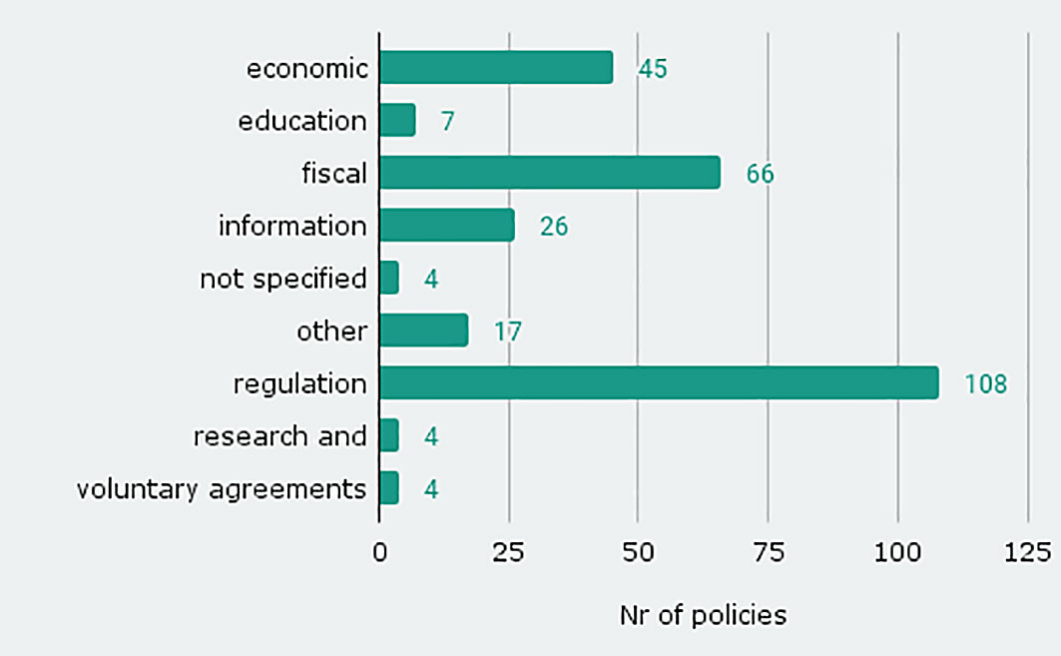
Number of policy instruments by instrument types.

The database includes existing and proposed **policy instruments** of all types as categorised by UNFCCC. ^32^ Most of the policy instruments in the database are of regulatory nature (108), economic (45, e.g. taxation) or fiscal (66, e.g. infrastructural or subsidies) (see Figure 3). The high share of regulatory instruments is in contrast to sufficiency policy instruments that are implemented or planned by EU member states, as listed in NECPs ^2^ where regulatory instruments are underrepresented. The diversity of instrument types shows that sufficiency policies are not only information campaigns leaving the responsibility to the individual, but a comprehensive mix of instruments. Sufficiency policy can thus be regarded as a policy field comparable to energy efficiency or renewable energies.

**Figure 4.**
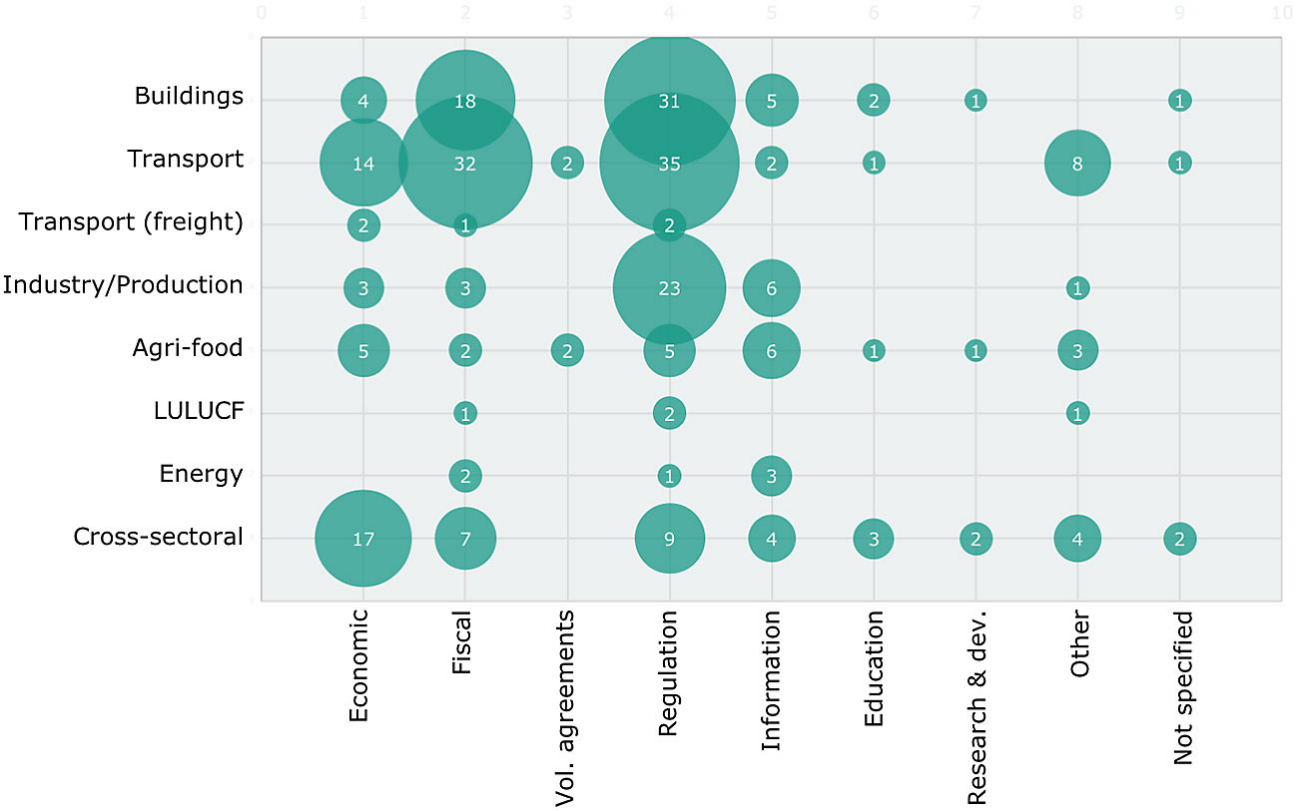
Number of policy instruments by sector and instrument type. Note: Bubble sizes indicate the number of instruments.

Figure 4 combines the information on sectors and instrument types. This highlights that the sufficiency policies proposed in the various sectors diverge in focus. In the building sector, fiscal (subsidy) instruments and regulations prevail while for the transport sector, the focus is more on fiscal/infrastructure policies, financial/tax incentives and regulation. A possible explanation for the prevalance of fiscal instruments are limits/biases of the underlying literature, as economic models and scenarios are driven by price assumptions. For the industry/production sector, mostly regulatory instruments are included while cross-sectoral policy instruments are mostly financial incentives through taxation and subsidies as well as regulation. For the other sectors, fewer instruments of all types are included.

As a step towards model inclusion of sufficiency policies, we searched for specific target **indicators** that may eventually be included into models or, vice versa, that models may potentially give as outputs to quantify energy sufficiency. The database includes 79 unique indicators such as e.g. ‘durability of products’, ‘car ownership rate’, ‘kg of exported meat’ and ‘ $m^{2}$ living space/person’ or ‘ $m^{2}$ unused living space’. For a complete list, see underlying data. These are indicators identified from the policy descriptions and may function as linking variables to sector and energy models and climate scenarios. ^36^

By summarizing the overall goals all identified sufficiency policy instruments, we identify 45 different **policy goals** (See underlying data). ^31^ While most are quite sector-specific and concrete such as reduce motorized individual transport or efficient use of living space, some goals, especially the ones of cross-sectoral policy instruments, aim at more general goals of human well-being e.g. equal society,alternative welfare indicators and that would involve more fundamental changes of organizing principles of societies e.g. re-distribute and reduce paid work-time or promote commons. This shows that demand-side options are not only a means to mitigate climate change, but can also involve a more general strategy to reach socio-ecological goals and quality of life.

## Discussion

Potentially, much like with energy efficiency and renewable energy policy, sufficiency policies are most effective and unfold all advantages, if not reduced to single measures but integrated into a consistent policy framework. We consider it still helpful to depict each policy instrument in detail in such a database on the one and provide the heterogeneity of sufficiency policy on the other side.

The database published along with this article is a first version and proof of concept. So far, we have a strong focus on European and German sources. We do not claim completeness and since it is a dynamic field, additional policy instruments will be added. We will also invite external persons to propose additional policies and plan to team up with other research stakeholders in the energy sufficiency field at German and EU level to investigate options for a successful continuation of the database including potential future (co-)hosts and possibilities for maintenance. Regarding its format, we plan to implement the sufficiency policy database as an online database.

Further planned developments of the database are the inclusion of quantitative effects (energy demand & GHG-reduction potentials) of the policies. To this end, policy impact chains need to be investigated and the policy representation in models be expanded to include them in ex-ante assessments and scenario studies. ^37^ Another possible addition is a prioritization regarding maturity, replicability and impact. ^38^ To make more transparent which policies are actually reducing barriers for sufficiency, we will add the categorisation of barrier-reduction and sufficiency-supporting. Furthermore, we will add good practice and implementation examples to the entries of the database.

In the field of barriers, more research is required to identify current political barriers which might prevent the implementation of sufficiency. Due to the partly fundamental character of the changes some policy instruments of sufficiency represent, further research on economic effects, macroeconomic dynamics and culture is required which is beyond the scope of the database itself but is fundamental for a successful implementation of sufficiency policy.

## Conclusion

Our energy sufficiency policy database of currently 281 policy instruments can serve as a basis to develop policy recommendations in the so far underrepresented field of sufficiency policy. Moreover, it provides a starting point for further research on sufficiency and climate change mitigation policies, especially for modelling and scenario studies. The identification of indicators is a first step to include sufficiency into modelling and be able to assess the effect of the different policy measures.

As the variety of different instrument types, goals and policy levels shows, sufficiency is a diverse policy field with multiple options of implementation and effect. The database encompasses specific instruments but also fundamental structural changes of societal organisation principles. Our interdisciplinary approach is helpful to cover the heterogeneity of sufficiency. The description on concrete information on sufficiency as policy, with goals, instruments and indicators as an interface to modelling can help to overcome existing barriers on sufficiency policy implementation. Making it more tangible can support its path to becoming a key strategy for energy policy.

## Data availability

Underlying data Zenodo: Sufficiency Policy Database DOI ^31^: *This project contains the following underlying data:* Sufficiency Policy Database

Source data https://energysufficiency.de/policy-database/ (latest version of the sufficiency policy database)

Data are available under the terms of the Creative Commons Attribution 4.0 International (CC BY 4.0).

## Author contributions

Benjamin Best: *Conceptualization, Funding Acquisition, Investigation, Methodology, Project Administration, Writing – Original Draft Preparation, Writing – Review & Editing*; Johannes Thema: *Conceptualization, Formal Analysis, Investigation, Methodology, Visualization, Writing – Original Draft Preparation, Writing – Review & Editing*; Carina Zell-Ziegler: *Conceptualization, Formal Analysis, Investigation, Methodology, Visualization, Writing – Original Draft Preparation, Writing – Review & Editing*; Frauke Wiese: *Conceptualization, Funding Acquisition, Investigation, Methodology, Project Administration, Writing – Original Draft Preparation, Writing – Review & Editing*; Jonathan Barth: *Resources*; Stephan Breidenbach: *Resources*; Leonardo Nascimento: *Resources, Writing – Review & Editing*; Henry Wilke: *Resources*

## Footnotes

^1^
For more details and examples see Zell-Ziegler *et al.* ^2^

^2^
EnSu - Research Group on energy sufficiency, https://energysufficiency.de/.
