# Supplementary material for: Building a database for energy sufficiency policies
Source: F1000Res. 2022 Jul 4;11:229. Originally published 2022 Feb 24. [Version 2] doi: 10.12688/f1000research.108822.2 (PMC9010800; doi:10.12688/f1000research.108822.2)
Supplement: Peer review history

# Peer review: Reviewer response for version 1

**DOI:** 10.5256/f1000research.120251.r127903

**Published:** 2022-04-14

**Authors:** Mirko Schäfer, Celia Burghardt

**Affiliations:** 1 INATECH, University of Freiburg, Freiburg, Germany; 2 INATECH, University of Freiburg, Freiberg, Germany

The authors present a database for sufficiency policies, including and categorising almost 300 policy instruments, with a focus on Germany and Europe.

 Sufficiency measures are currently underrepresented in the discussion of transformation pathways towards a climate neutral energy system. In particular, this applies to energy system modelling for scenario development and analysis. Often the focus is on the technological transformation and advancement, without the consideration of demand-side solutions. Although recently an increased interest in such demand-side solutions can be observed, there is still a research gap in identifying, quantifiying and modelling sufficiency strategies and policies. The authors address this issue by presenting a database for energy sufficiency policies.

 In the current form, the database already represents a valuable source of information about a large number of sufficiency policies, categorised in a very well structured way and linked to references. In this way, already in its present form, it can be seen as an important starting point and gateway for anyone interested in sufficiency policies. The audience already more familiar with such concepts might object to certain categorisation choices or seek additional information, but also for this purpose the database can be helpful as a seed and potential platform for discussion.

 Nevertheless, in its current form, the database only represents a starting point for further research. This is clearly acknowledged by the authors in the article and here is not intended as an argument against the publication of this contribution. Very important next steps could be towards more quantification (parameter settings as an input for system modelling), impact chains as a guidance for sector-specific modelling, and the extension beyond Europe.

 To allow further development, a clearer view on how the community can interact with and through the database should be developed. This should include both the ability for users to contribute to the database (facilitated by clear explanation and examples on the categorisation) and the possibility to provide feedback on the usefulness or shortcomings of the database. For this purpose, it might be an option to link the database to a (moderated) discussion forum.

 With regard to the analysis of the already represented sufficiency policies, it would be interesting to discuss in more detail why certain types of instruments in certain sectors are dominant in the database. Is this due to limited options for other sectors/instruments, or can this be explained with a bias in the underlying research?

 To conclude, we are confident that the database serves as a valuable source for the audience seeking a first introduction into sufficiency policies, as well as an important starting point for research towards a broader and deeper integration of sufficiency policies into energy system analysis and modelling.

Is the work clearly and accurately presented and does it cite the current literature?

Yes

If applicable, is the statistical analysis and its interpretation appropriate?

Not applicable

Are all the source data underlying the results available to ensure full reproducibility?

Yes

Is the study design appropriate and is the work technically sound?

Yes

Are the conclusions drawn adequately supported by the results?

Yes

Are sufficient details of methods and analysis provided to allow replication by others?

Yes

Reviewer Expertise:

Energy system analysis and modelling

We confirm that we have read this submission and believe that we have an appropriate level of expertise to confirm that it is of an acceptable scientific standard.

# Response

**Published:** 2022-06-30

**Authors:** Benjamin Best

Dear Mirko, thank you for your review! It is very valuable to us for the next versions of our database. We thought about possible explanations why some instrument types are dominant in sectors. A possible reason is that price instruments are easiest to model, so that we need to consider a certain bias due to the methods of the underlying research. This will help to consider limitations when drawing conclusions from analysis of our database.

# Peer review: Reviewer response for version 1

**DOI:** 10.5256/f1000research.120251.r125368

**Published:** 2022-03-24

**Authors:** Gibran Vita

**Affiliations:** 1 Environmental Sciences, Open University of the Netherlands, Heerlen, The Netherlands

The authors make a very good effort and praiseworthy initiative to try to bring sufficiency into more concrete policy terms and instruments. The database is a living document. As such, it cannot be currently judged as a finished product. It will change shape as it progresses.

 Its current version does seem to over-rely or over-represent few sources and countries (Germany). In that case, I don´t see it as a really comprehensive database. Too many countries and initiatives missing, even in Europe. It is however remarkable that with such a narrow range of sources you still find more than 100 policies. Maybe a full-blown European database would be massive, but highly desirable in my opinion.

 One of the sources is “expert consultation” but no transparency on this as far as I could read.  Maybe this was just meant to get an overview of current databases.

 In its current form, it seems more like a summarized and user-friendly way to navigate a handful of reports. Which is still useful, especially since you translated German documents into English entries, but not really what the title and abstract seems to sell. I look forward for it to grow and become more diverse and comprehensive.

 Concrete suggestions:

 The database could benefit from consistency in reporting which countries the policies are present in. In some categories the countries are mentioned, in some others not, even when a given policy seems to only apply to Germany, for example. Would search by country be enabled?

 Another interesting variable to include would be the broader policy landscape or directive where the particular policy or instrument stems from. EU directive, Paris agreement, SDGs, UN biodiversity, etc. As the purpose of the database is to understand how to promote sufficiency policy, it seems key to understand which kind of broader policy movements facilitated that particular industry.

 The base year would be relevant to mention.

 You can abbreviate the Shift, Avoid, to save space and include other things.

 The title of the database should probably include "Europe" in the title. Or even Germany. Whatever you think represents it best.  The sector “LULUCF”  (and maybe some food and agriculture) doesn’t seem to fit the “energy” exclusive topic.

 In that sense, I would think that an indication of which resource is mainly spared: energy, carbon, etc. Or if it’s synergistic measure: land, water, energy, etc. I understand this would be much more work at this point as it would have been to consider it since the start.

 Perhaps a more critical point: to make it more useful it would be helpful to have the “implementation challenges” or “complementary policies” to avoid loss of wellbeing or energy backfire. For example, ban of domestic flights might rise car demand if we don’t have a massive train subsidy. Challenges of reducing meat would need more education on plant-based lifestyles or a ban on artificial chemicals of plant-based meat substitutes. We need to think from the policy-maker viewpoint.

 Would have been useful to breakdown the biggest categories: regulation, fiscal and economic into subcategories. For example “regulation: ban”. All these are very broad. For example, if one is interested on looking at bans. The search bar could be used instead but not sure if it’s working properly or if it’s also looking in the reference document? For example, if I write “ban” I get some results but can't find the word “ban” in those entries.

 What is the plan to make it grow and have a life of its own? There doesn’t seem to be a way to submit entries. Most importantly, having experience in these kinds of projects I would not rely on the good will of people to submit entries.

 I strongly suggest the authors to consider a massive crowd-sourcing experiment where you extend authorship to many many more researchers and practitioners who are on top of this topic, maybe in exchange for min 5-10 entries. There is no limit to how many authors a database or paper can have. Might be a worthwhile experiment.

 Great initiative and wish you success,

 Gibran Vita

Is the work clearly and accurately presented and does it cite the current literature?

Yes

If applicable, is the statistical analysis and its interpretation appropriate?

Not applicable

Are all the source data underlying the results available to ensure full reproducibility?

Yes

Is the study design appropriate and is the work technically sound?

Yes

Are the conclusions drawn adequately supported by the results?

Yes

Are sufficient details of methods and analysis provided to allow replication by others?

Partly

Reviewer Expertise:

Industrial Ecology

I confirm that I have read this submission and believe that I have an appropriate level of expertise to confirm that it is of an acceptable scientific standard.

# Response

**Published:** 2022-06-30

**Authors:** Benjamin Best

Thank you for your valueable feedback. We will update our database and consider your feedback for the next version. Your review provides us with actionable guidance for the next steps.
